# Laparoscopic cholecystectomy based on Laennec approach via the cystic plate with lymphadenectomy in Calot's triangle for gallbladder neoplasms: Initial experience and technical details

**DOI:** 10.1016/j.iliver.2023.10.001

**Published:** 2023-10-18

**Authors:** Bin Ouyang, Laizhu Zhang, Yajuan Cao, Zhongjie Xing, Jin Peng, Yang Yue, Decai Yu

**Affiliations:** a Nanjing Central Hospital, Nanjing 210008, China; b The Affiliated Drum Tower Hospital, Nanjing University Medical School, Nanjing 210008, China

**Keywords:** Laennec approach, Cystic plate, Laparoscopic cholecystectomy, Gallbladder neoplasms, Patient safety

## Abstract

**Background:**

It is still challenging to define the exact stage of early gallbladder carcinoma with preoperative imaging. Generally, subserous gallbladder is dissected for the potential early gallbladder carcinoma, which may cause incomplete tumor resection or tumor spread especially for the patients with T2 stage. Here, we reported our experience and safety of Laennec approach via the cystic plate to dissect the whole gallbladder with lymphadenectomy in Calot's triangle for accurate diagnosis and stage in gallbladder neoplasms.

**Methods:**

The anatomical gap between Laennec capsule and the cystic plate serves as the landmark to dissect the whole gallbladder through Laennec approach. Laparoscopic cholecystectomy based on Laennec approach via the cystic plate, together with lymphadenectomy in Calot's triangle, was performed in 17 patients with gallbladder neoplasms.

**Results:**

All patients had less intraoperative bleeding, no gallbladder breakage, no bile leakage, and accurate intraoperative rapid pathological staging under the corresponding strategies. The duration of surgery was comparable to that of traditional laparoscopic cholecystectomy.

**Conclusion:**

Laparoscopic cholecystectomy based on Laennec approach via the cystic plate, together with lymphadenectomy in Calot's triangular is safe for gallbladder neoplasms. In the future, the prospective clinical trial is going on to confirm the feasibility and effectiveness of this approach.

## 1 Introduction

Gallbladder cancer (GBC) is the sixth common malignancy of the digestive tract with poor prognosis when diagnosed at an advanced stage [1]. Early diagnosis and precise therapy of GBC are particularly important for prognosis [2]. The Guidelines for the Diagnosis and Treatment of GBC states that Stage T3 or T4 can be defined accurately with preoperative imaging, such as enhanced multilayer spiral CT or MRI, therefore the patients undergo the corresponding surgical protocol [3]. However, it is challenging to differentiate stage T1 and T2 for GBC with radiological examination, even that sometimes the preliminary T stage can be ascertained by imaging studies [4,5]. Therefore, surgeons have to seek intraoperative frozen pathology to identify the tumor nature and invasion depth of gallbladder neoplasms (GBN) after laparoscopic cholecystectomy [6,7]. Generally, subserous cholecystectomy is performed, which has the potential risk to cause incomplete tumor resection, tumor spread, or inaccurate stage for the patients with T2 stage [7,8]. Moreover, the patients with T1b or T2 stage tumors are recommended for expanded radical resection to improve survival prognosis [8,9].

The gallbladder has 3 layers, including mucosa, muscularis, and serosa/adventitia. In the gallbladder fossa, adventitia is composed of collagen, elastic tissue, fat, vessels, lymphatics and nerves, which continues as the cystic plate. Generally, this adventitia with the cystic plate is left in the gallbladder bed after subserous cholecystectomy. It has been previously confirmed that Laennec capsule, surrounding all the parenchymal, is free from the visceral peritoneum, adventitia and cystic plate, and inferior vena cava outside the liver, and extends into liver along with the hepatic pedicle and out liver with hepatic veins [10]. In 2018 Yu's term proposed that Laennec capsule could be used as a landmark for anatomical hepatic resection, guiding extrahepatic isolation (perihepatic and retrohepatic isolation) and intrahepatic isolation (hepatic pedicle and hepatic vein isolation), which was nominated as the Laennec approach for hepatectomy [11]. There is the gap between adventitia/the cystic plate and Laennec capsule closing the parenchymal, which may serve as the landmark to dissect the gallbladder from the gallbladder bed.

**Fig. 1:**
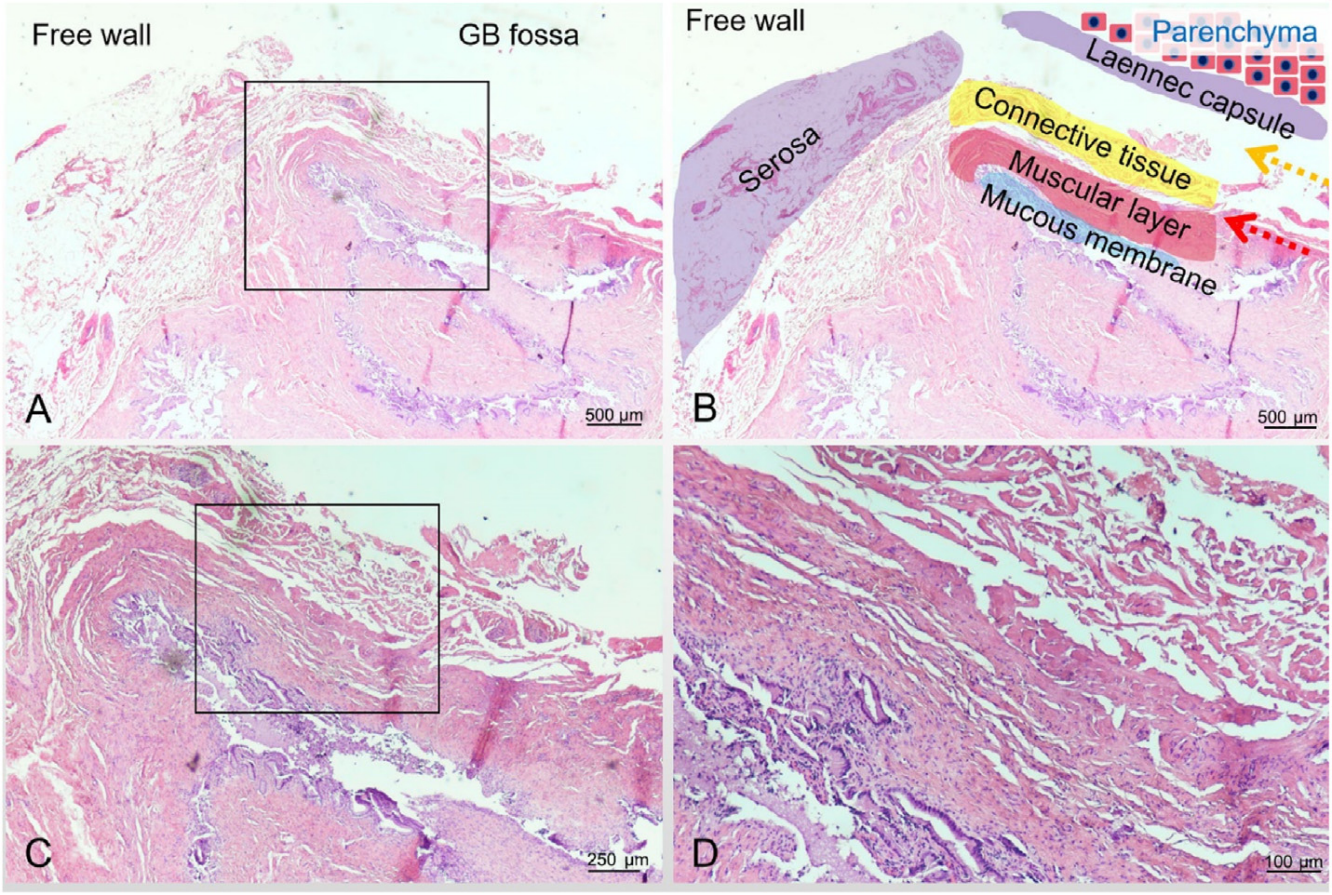
The representative HE sections in the junction between gallbladder fossa and free wall of patient 3. In Fig. A the junction of the gallbladder bed and the serosa of the gallbladder was presented under a 20× macro lens. The gallbladder fossa is mainly divided into three layers, as marked with different color zone in Fig. B, from the inside to the outside are the mucous membrane, muscular layer and connective tissue (adventitia). Traditional approach for cholecystectomy, marked with red arrow, were performed along muscular layer, while Laennec approach, marked with yellow arrow, were performed along Laennec capsule closing to adventitia. Figures C and D were presented under a 40× and 100× macro lens from the area marked in the box in Figs A and C, respectively.

Therefore, the reasonable protocol, Laennec approach via the cystic plate for laparoscopic cholecystectomy in Fig. 1, was designed for the potential early GBC. The protocol, including gallbladder dissected completely through the Laennec gap between adventitia/the cystic plate and Laennec capsule together with lymphadenectomy in the Calot's triangle, expands the scope and depth of resection, further avoids the risk of tumor destruction, and contributes to the accurate staging of the potential GBC, which in turn allows for timely remedial surgery according to the guidelines. The initial experience and technical details were described as follows.

## 2 Methods

### 2.1 Study design

This study was performed in Nanjing Central Hospital and Drum Tower Hospital. The protocol was approved by the Research Ethics Committee of Nanjing Central Hospital (2021NJCHLL003) and Drum Tower Hospital (2020-310-02), and conformed to the ethical guidelines of the 1975 Declaration of Helsinki [12]. Informed consents were obtained in writing from each patient. Since September 2020, patients with potential early GBC have been enrolled in this trial. The preoperative diagnosis was dependent on ultrasonography and MRI with contrast. Patients with more than T3 GBC were excluded. Some basic information of eighteen patients was summarized in Table 1.

**Table 1:** Basic information of the patients in this trial.

Number	Gender	Age	Diagnosis	Date	Location	Tumor size (cm)
**No. 1**	Female	60	gallbladder polypoid with stone	2020/9/26	Fundus	0.8 × 1.7
**No. 2**	Male	55	gallbladder polypoid with stone	2021/6/11	Fundus	1.3 × 1.8
**No. 3**	Female	70	gallbladder polypoid	2021/7/30	Neck	1.1 × 2.4
**No. 4**	Female	57	gallbladder polypoid	2021/10/8	Body	1.1 × 1.5
**No. 5**	Male	45	gallbladder polypoid	2021/10/17	Body	1.0 × 1.6
**No. 6**	Male	66	gallbladder adenocarcinoma	2021/10/19	Fundus	1.6 × 3.2
**No. 7**	Male	30	gallbladder polypoid with stone	2022/2/18	Body	1.4 × 0.9
**No. 8**	Female	62	gallbladder polypoid	2022/5/29	Body	1.0 × 0.6
**No. 9**	Female	57	gallbladder polypoid with stone	2022/7/12	Body	1.0 × 0.7
**No. 10**	Female	40	gallbladder polypoid	2022/7/26	Body	1.0 × 0.6
**No. 11**	Female	64	gallbladder adenocarcinoma	2022/8/8	Fundus	3.2 × 1.6
**No. 12**	Male	47	gallbladder polypoid with stone	2022/9/15	Body	1.1 × 1.2
**No. 13**	Female	55	gallbladder polypoid with stone	2022/10/8	Body	1.5 × 0.9
**No. 14**	Female	39	gallbladder polypoid with stone	2022/10/28	Body	1.0 × 0.9
**No. 15**	Male	35	gallbladder polypoid	2022/11/2	Body	1.2 × 0.5
**No. 16**	Male	27	gallbladder polypoid with stone	2022/11/18	Body	1.5 × 0.8
**No. 17**	Female	46	gallbladder polypoid	2022/12/7	Body	1.1 × 0.5

### 2.2 Procedures

The patient's position and trocar layout were similar to those of traditional laparoscopic cholecystectomy [13]. The patient was placed on the table in the supine position. After induction of general endotracheal anesthesia, the abdomen was sterilely prepped and draped. A 10 mm incision was made beneath the umbilicus for laparoscope. A pneumoperitoneum of 15 mmHg (1 mmHg = 0.133 KPa) was established. Diagnostic laparoscopy was then performed to check the tumor invasion or metastasis. Then 12 mm trocar as the operating port was placed in the xiphoid position, followed by a 5-ram trocar to retract the fundus of the gallbladder upward in the fight lower abdomen. A 5-mm trocar was placed as the first assistant port in the fight subcostal position.

All patients underwent laparoscopic cholecystectomy via the Laennec approach with lymphadenectomy in Calot's triangle, which is described in Fig. 3 and supplemental video 1. To check out metastasis through abdominal exploration, dissect the ventral visceral peritoneum of Calot's triangle along the common bile duct (Fig. 3A) and the dorsal visceral peritoneum along the right hepatic pedicle (Fig. 3B), separate and detach the gallbladder artery and gallbladder duct close to the common bile duct, dissect lymph nodes in Calot's triangle (Fig. 3C), dissect the visceral peritoneum of the liver close to the gallbladder bed with an electrocoagulation hook, bluntly separate into the Laennec gap, detach the gallbladder plate (Fig. 3D), and completely strip the whole gallbladder, including adventitia, through the Laennec approach (Fig. 3E–G). The gallbladder bed is rinsed and hemostated tightly, and bile leakage is carefully examined (Fig. 3H). The gallbladder was opened to confirm the tumor site and the depth of the invasion and to be sent for frozen pathology. During laparoscopic cholecystectomy based on the Laennec approach, the cystic plate was also dissected lastly (Fig. 4). All procedures were performed by the same attending surgeon (Decai Yu).

Furthermore, the samples, including gallbladder and lymph nodes, were sent to the frozen pathological examination. All patients with benign neoplasms recovered after the frozen pathological examination, while the other patients with malignant neoplasms underwent the corresponding strategy according to the stage.

Procedure evaluation and follow up.

The procedure relevant index, such as frozen pathological results, duration for the key steps, estimated blood loss, bile leakage, morbidity and mortality, were collected. All patients were followed up for six months for morbidity, mortality, and tumor recurrence.

## 3 Results

**Fig. 2:**
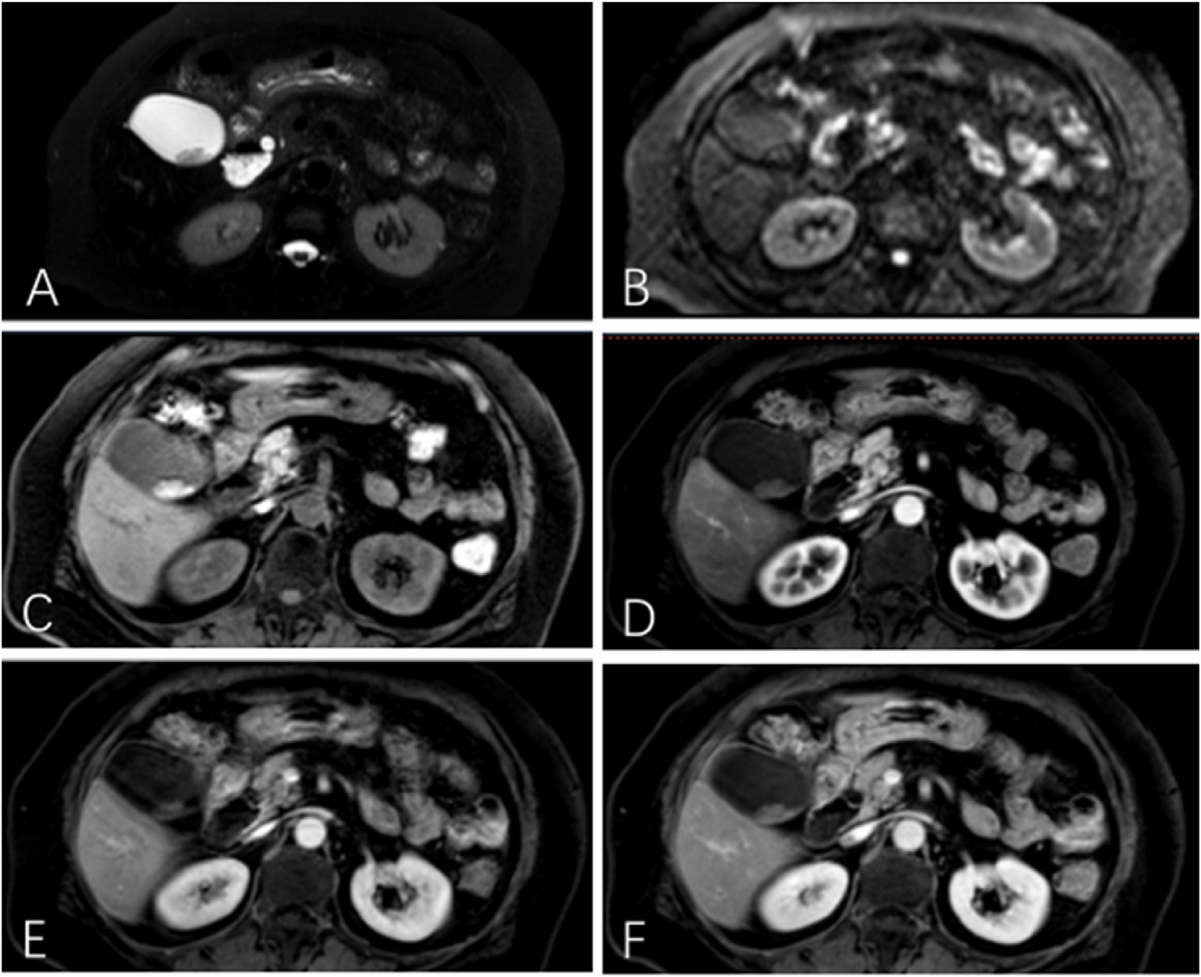
MRI features of gallbladder adenoma from Patient No 3. Axial T2-weighted (A) and T1-weighted (C) images show marked asymmetric mural thickening that is moderately hyperintense without diffusion restriction (B), and shows poor contrast enhancement in artery, portal, and vein phase (D–F). The tumor size was 2.4 cm times 1.1 cm.

**Fig. 3:**
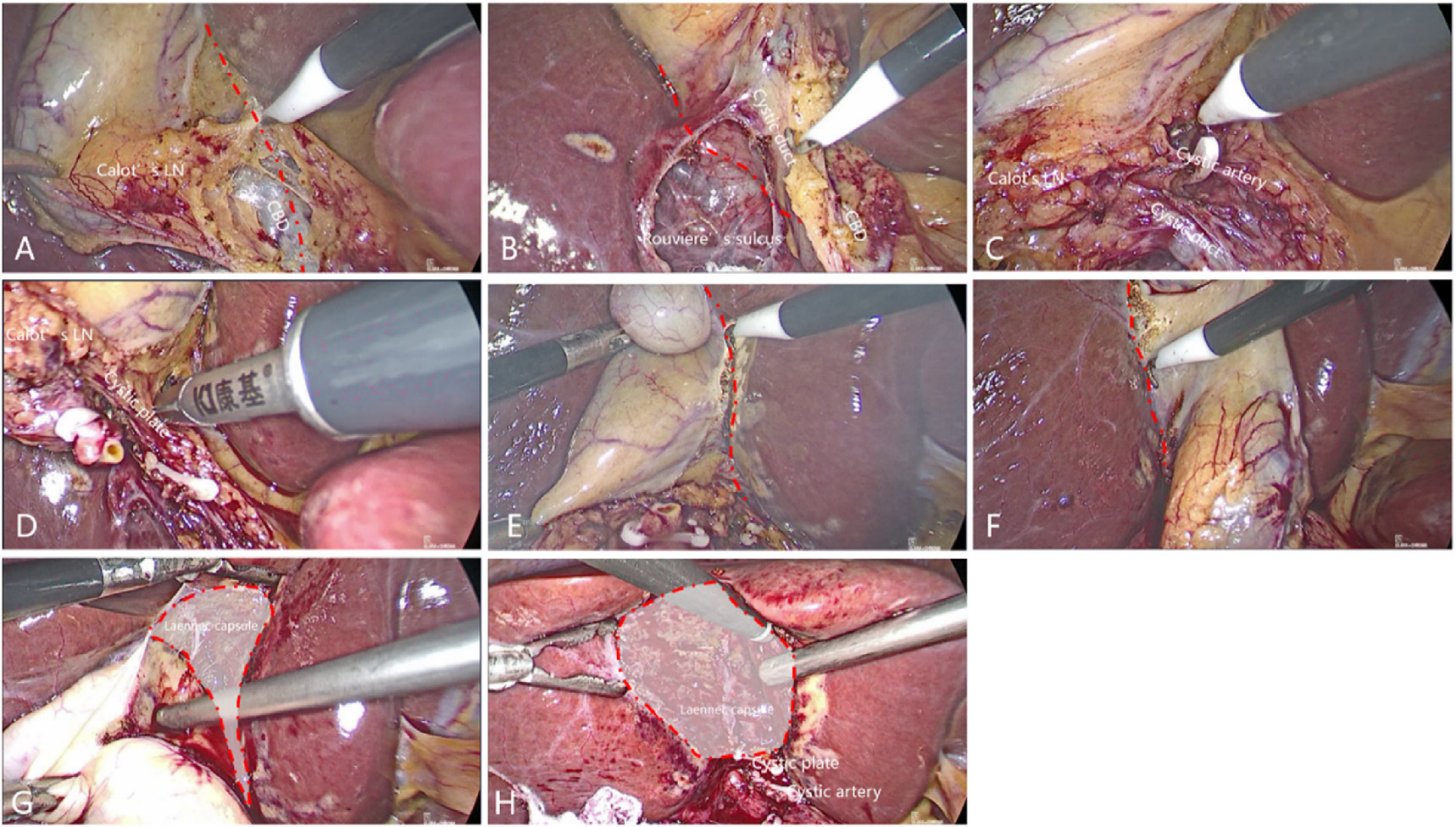
Procedures for laparoscopic cholecystectomy based on Laennec approach with cystic plate dissected first for patient No. 3. Firstly, dissect the ventral visceral peritoneum of the Calot's triangle along common bile duct (A) and the dorsal visceral peritoneum along the right hepatic pedicle (B), separate and detach the gallbladder artery and gallbladder duct (C), then dissect the visceral peritoneum of the liver close to the gallbladder bed with an electrocoagulation hook, bluntly separate into the Laennec gap, detach the gallbladder plate (D), and completely strip the gallbladder from the gallbladder bed through the Laennec approach (E–G), the gallbladder bed is rinsed and tightly hemostated, and bile leakage is carefully examined (H). The red line was marked to guide the dissection path. The shadow zone was coved by Laennec capsule.

**Fig. 4:**
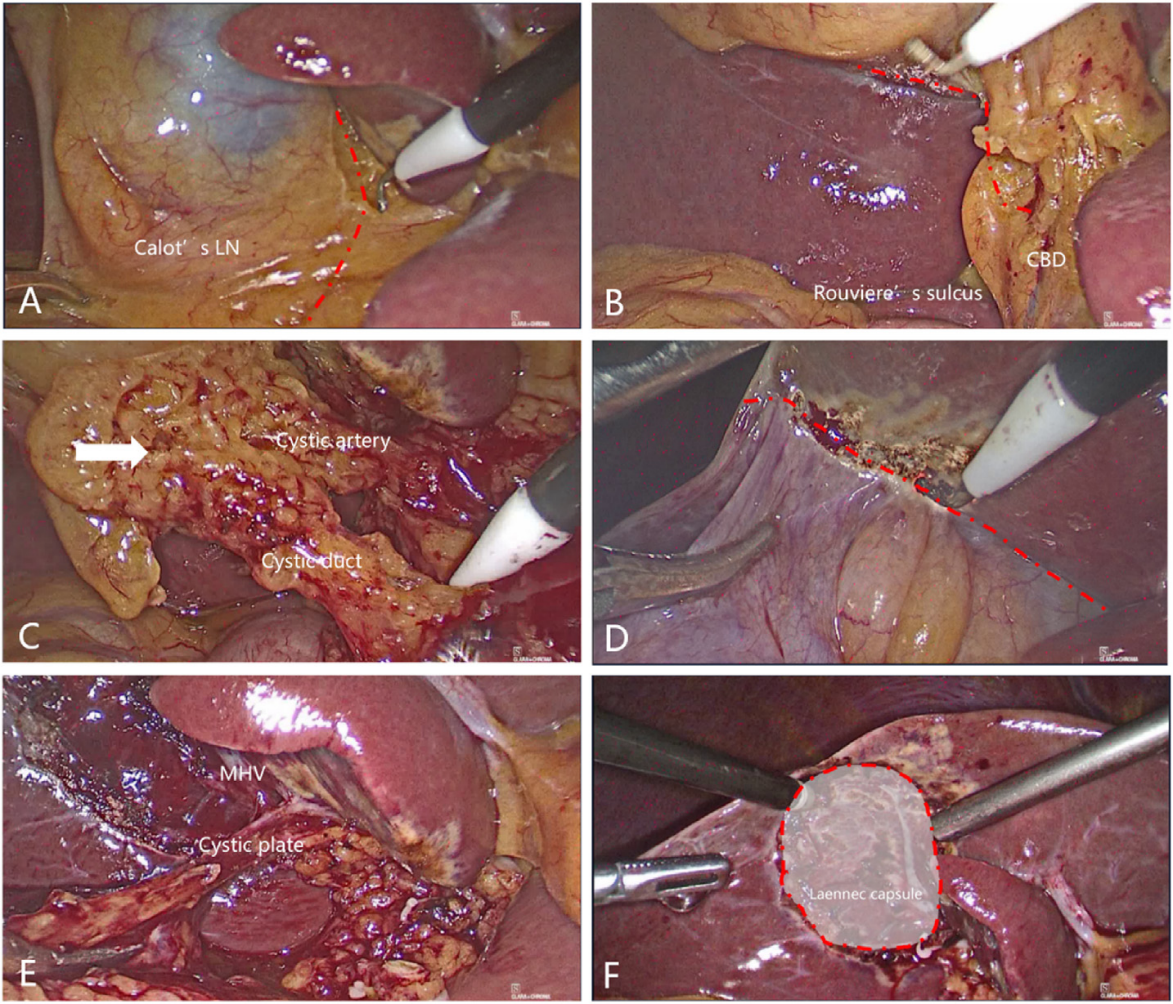
Procedures for laparoscopic cholecystectomy based on Laennec approach with cystic plate dissected lastly for patient No. 5. First dissect the ventral visceral peritoneum of the Calot's triangle along common bile duct (A) and the dorsal visceral peritoneum along the right hepatic pedicle (B), separate and detach the gallbladder artery and gallbladder duct (C). Then dissect the visceral peritoneum of the liver close to the gallbladder bed with an electrocoagulation hook, bluntly separate into the Laennec gap, and completely strip the gallbladder from the gallbladder bed membrane through the Laennec approach (D–E), detach the gallbladder plate (E), the gallbladder bed is rinsed and tightly hemostated, and bile leakage are carefully examined (F). The red line was marked to guide the dissection path. The shadow zone was coved by Laennec capsule. CBD means common bile duct; MHV means middle hepatic vein; LN means lymph node.

Since September 2020, seventeen patients were enrolled into this study. Among them including 7 men and 10 women, aged 27–70 years old, the preoperative diagnosis was gallbladder neoplasm. Ultrasonography and MRI with contrast showed that the tumor size ranged from 1.0 cm to 3.2 cm, of which 8 cases had gallbladder stones, and usually had occasional right upper quadrant vague pain discomfort, and the remaining 5 cases had no obvious clinical symptoms. Figure 1 illustrated the anatomical hierarchy of laparoscopic cholecystectomy via the Laennec approach. The representative MR images were presented in Fig. 2 for Patient No 3. All key index was summarized in Table 1. Seventeen patients, as the subjects with potential early GBC, underwent laparoscopic cholecystectomy based on Laennec approach via the cystic plate with Calot's triangle lymph node dissection, which were described in Fig. 3 and supplemental video 1 with cystic plate dissected first, and in Fig. 4 and the supplemental video 2 with cystic plate dissected lastly. The procedure-relevant indexes of the patients in this trial are summarized in Table 2.

**Table 2:** Procedure relevant index of the patients in this trial.

Number	Pathological diagnosis	Shift strategy	Blood loss (mL)	Bile leakage	Duration of surgery (mins)	Morbidity (Clavien-Dindo Classification)	Hospital stay (days)
**No. 1**	Inflammatory polyps	No	10	No	55	Grade I	3
**No. 2**	Cholesterol polyps	No	20	No	50	Grade I	3
**No. 3**	Adenoma	No	10	No	60	Grade I	3
**No. 4**	Adenoma	No	20	No	45	Grade I	3
**No. 5**	T1b Adenocarcinoma	Portal lymphadenectomy	30	No	120	Grade I	5
**No. 6**	T2a Adenocarcinoma	S4b/5 wedge resection with portal lymphadenectomy	100	No	235	Grade I	13
**No. 7**	Cholesterol polyps	No	50	No	85	Grade I	2
**No. 8**	Cholesterol polyps	No	10	No	40	Grade I	3
**No. 9**	Cholesterol polyps	No	10	No	35	Grade I	2
**No. 10**	Cholesterol polyps	No	10	No	50	Grade I	7
**No. 11**	Adenocarcinoma	Right hemihepatectomy	300	Yes	320	Grade I	19
**No. 12**	Cholesterol polyps	No	10	No	100	Grade I	2
**No. 13**	Cholesterol polyps	No	10	No	40	Grade I	3
**No. 14**	Cholesterol polyps	No	5	No	40	Grade I	2
**No. 15**	Cholesterol polyps	No	10	No	100	Grade I	2
**No. 16**	Inflammatory polyps	No	10	No	45	Grade I	2
**No. 17**	Cholesterol polyps	No	20	No	45	Grade I	2

The frozen pathological examination showed that two cases were adenoma, ten cholesterol polyps, two Inflammatory polyps, and three adenocarcinomas. Fourteen patients with benign neoplasms recovered after the frozen pathological examination. Patient No. 5 with GBC without lymph node metastasis (T1b) underwent the shift strategy with portal lymphadenectomy under laparoscopy. Patient No. 6 with GBC with Calot's lymph node metastasis, defined as stage IIIB (T2a, N1, M0), underwent the shift strategy with S4b/5 wedge resection with portal lymphadenectomy and Roux-en-Y hepaticojejunostomy under laparoscopy. In addition, patient No. 11 with GBC with liver invasion and visible vascular tumor thrombus, defined as stage IIIA (T2, N0, M0), underwent the shift strategy with right hemihepatectomy under laparoscopy.

All procedures were successfully completed under laparoscopy, and the operation time was 35–320 min with estimated blood loss from 5 mL to 300 mL. The drainage tube was placed after the operation in three patients with adenocarcinoma. Only one patient has occurred complication, such as bile leakage. Fourteen subjects with benign neoplasms were discharged on POD 3 for the clinical trial. The patients undergoing portal lymphadenectomy were discharged on POD 5, while the patients undergoing radical resection were discharged on POD 13 and 19. There was no recurrence on the patients with adenocarcinoma after six months.

## 4 Discussion

Laparoscopic cholecystectomy based on Laennec approach via the cystic plate was performed in seventeen patients with GBN. The operative index with this approach, such as blood loss and operation duration, compared to traditional laparoscopic cholecystectomy. There was no morbidity or mortality. Moreover, all neoplasms were evaluated accurately and coped with the corresponding protocol according to the guideline for GBC. During follow-up in half a year, there was no recurrent for the patients with adenocarcinoma. Therefore, laparoscopic cholecystectomy based on Laennec approach via the cystic plate was safe and feasible to define the nature and invasion of GBC.

Membranes are anatomical barriers that separate tissues from organs and form the anatomical gap with adjacent organs or tissues, which provides the natural path for surgeons to dissect. The concept of membranous surgery based on membrane structure gradually takes shape [14]. Laparoscopic cholecystectomy via Laennec approach was performed under the guidance of natural gap between Laennec capsule and the adventitia/cystic plate of the gallbladder. With blunt separation, there were very few branches between gallbladder and liver to be dissected. Membranous anatomy was helpful us to define the surgical level “Holly Plane”, which facilitates the promotion and standardization of surgical protocols. And it is also conducive to radical tumor resection and improves prognosis [15]. We used the suction to keep the field clear, then divest the gallbladder from the bed for about 10 min. In this study, rapid pathologic diagnosis of suspected early-stage gallbladder cancer during routine resection accounted for 17.6%. Three cases were confirmed by intraoperative frozen section and postoperative pathology. All cases underwent radical resection, which were dissected safely without morbidity; One patient with GBC adenocarcinoma without lymph node metastasis (T1b) underwent the shift strategy with portal lymphadenectomy under laparoscopy, while one patient with GBC with Calot's lymph node metastasis (T2a) underwent the shift strategy with S4b/5 wedge resection with portal lymphadenectomy and Roux-en-Y hepaticojejunostomy under laparoscopy. Furthermore, one patient with GBC without lymph node metastasis, defined as stage IIIA (T2, N0, M0), underwent the shift strategy with right hemihepatectomy under laparoscopy. Through this approach, the whole gallbladder was dissected to keep the tumor complete, accurately evaluate tumor stage, prevent tumor spread, and select the corresponding protocol, especially for the patients with T1 or T2.

In addition, lymphadenectomy in Calot's triangle was also included in this protocol. The AJCC Cancer Staging Manual, Seventh Edition, subdivided lymph nodes of gallbladder cancer into two stations, and Calot's lymph node was included as the first station [16]. As we know, the rates of lymph node metastasis in T1a, T1b, and T2 patients were up to 7%, 11.1%, and 44.3%, respectively [17]. The actual prevalence of lymph node metastasis was much high after adjusting for the probability of missing nodal disease. It was clearly demonstrated that a relatively large proportion of patients with T1b and T2 GBC might suffer from an occult nodal disease [18]. In this study, the T stage of the patient with GBC was T2a with the positive Calot's lymph node was, while all other lymph nodes were negative. Therefore, it was important for surgeons to perform the lymphadenectomy in Calot's triangle, which was significant to define the accurate N stage and select the accurate the corresponding strategy according to the guideline of GBC.

This approach was based on the histology of gallbladder. Only seventeen patients were enrolled to testify the safety of this protocol for laparoscopic cholecystectomy at the current phase. Much many patients need to be testified for safety of the operation and feasibility of tumor stage, and long follow-up need to be testified for the efficiency for over survive. Therefore, we plan to start the perspective cohort study for the patients with potential GBC in our center, which will further testify the feasibility and effectiveness of this approach.

## Funding

The authors declare the following financial interests/personal relationships that may be considered as potential competing interests. Decai Yu reports financial support was provided by National Natural Science Foundation of China (ID: 82173129) and Nanjing Health Science and Technology Development Foundation (Grant No.YKK21244).

## Author contributions

Bin Ouyang, Laizhu Zhang, Yajuan Cao, Zhongjie Xing, Jin Peng, Yang Yue, and Decai Yu treated the patients. Bin Ouyang, Laizhu Zhang, Yajuan Cao, Zhongjie Xing, Jin Peng, and Yang Yue collected the epidemiological and clinical data. Bin Ouyang and Laizhu Zhang processed statistical data and drafted the manuscript. Decai Yu had the idea for and designed the study. Decai Yu revised the final manuscript and was responsible for summarizing all the data.

## Acknowledgments

We thank all the patients involved in this study.

## Declaration of competing interest

The authors declare that they have no known competing financial interests or personal relationships that could have appeared to influence the work reported in this paper.

## Data available statement

The datasets generated and analyzed during the current study can not be made available for public access because of the privacy of patients but can be obtained from the corresponding author on reasonable request if approved by the Ethics Committee of Nanjing Central Hospital and Drum Tower Hospital.

## Ethics statement

This study was performed in Nanjing Central Hospital and Drum Tower Hospital The protocol was approved by the Research Ethics Committee of Nanjing Central Hospital (2021NJCHLL003) and Drum Tower Hospital (2020-310-02), and conformed to the ethical guidelines of the 1975 Declaration of Helsink.

## Informed consent

Formal written informed consent was obtained from each patient and the work was carried out in accordance with the declaration of Helsinki.
